# Genetic variants in antigen presentation-related genes influence susceptibility to hepatitis C virus and viral clearance: a case control study

**DOI:** 10.1186/s12879-014-0716-8

**Published:** 2014-12-21

**Authors:** Peng Huang, Li Dong, Xiaomei Lu, Yuanyuan Zhang, Hongbo Chen, Jie Wang, Yun Zhang, Jing Su, Rongbin Yu

**Affiliations:** Department of Epidemiology and Biostatistics, School of Public Health, Nanjing Medical University, Nanjing, 211166 China; Department of Infectious Diseases, the People’s Hospital of Jiangsu Province, Nanjing, 210029 China; Department of Infectious Diseases, the Jurong People’s Hospital, Jurong, 212400 China; Department of General Practice, Kangda College, Nanjing Medical University, Nanjing, 210029 China; Department of Epidemiology, Medical Institute of Nanjing Army, Nanjing, 210002 China

**Keywords:** Hepatitis C, Genetic polymorphism, Human leukocyte antigen, Infection outcomes, Chinese population

## Abstract

**Background:**

Genes related to antigen presentation pathway, which are in the non-classical class-II region of human leukocyte antigen (HLA), play a vital role during the infection of hepatitis C virus (HCV).

**Methods:**

The current study determined the genotypes of 34 tagging-SNPs (single nucleotide polymorphisms) from 9 candidate genes (*HLA-DMA, HLA-DMB, HLA-DOA, HLA-DOB, TAP1, TAP2, LMP2, LMP7,* and *tapasin*) in a Chinese population of paid blood donors with high risk of HCV infection. The distributions of those SNPs were compared among the 1207 former paid blood donors with different HCV infection outcomes.

**Results:**

*HLA-DMA* rs1063478 and *HLA-DOA* rs2284191 were independent factors of acquiring HCV infection. Carrying three favorable alleles of rs1063478-T and rs2284191-G offered the highest protective effect (odds ratio = 0.46, 95% confidence intervals = 0.27-0.78). *HLA-DOB* rs7383287 and *LMP2* rs17587 were independent factors of infection chronicity. Subjects carrying two favorable alleles of rs7383287-G and rs17587-A had a decreased risk of HCV chronicity (odds ratio = 0.42, 95% confidence intervals = 0.26-0.66). The interaction analysis showed that experience of plasma donation interacted with the combined effects of rs1063478 and rs2284191 for HCV susceptibility, and the experience of whole blood donation interacted with the association of rs7383287 with HCV clearance.

**Conclusions:**

Our results suggested that genetic variants in antigen presentation pathway had influence on susceptibility to HCV infection and viral clearance. *HLA-DMA* rs1063478, *HLA-DOA* rs2284191, and *HLA-DOB* rs7383287 were identified as novel loci in Chinese population that were involved in HCV infection.

**Electronic supplementary material:**

The online version of this article (doi:10.1186/s12879-014-0716-8) contains supplementary material, which is available to authorized users.

## Background

Hepatitis C virus (HCV) infection is one of the most common chronic blood-borne infections in the world. The estimated HCV prevalence was 2.35% in 2010, affecting 160 million individuals, 29 million of whom were in China [1],[2]. One significant characteristic of HCV infection is its high possibility of chronicity, which usually leads to cirrhosis and hepatocellular carcinoma [3],[4].

The human leukocyte antigen (HLA) genomic region at chromosomal position 6p21 encodes many genes, which are important for immune system [5]. These genes are categorized into three basic groups: class I (*HLA-A*, *-B*, and *-C*), class II (*HLA-DR*, *-DQ*, and *-DP*), and class III. Previous studies have focused on classical class-I and -II regions and showed that polymorphisms in these regions were associated with chronic hepatitis C [6]–[8]. There are also some non-classic genes located among those classic regions and genetic functions of those genes are not fully understood yet. For example, *TAP* (the transporter associated with antigen processing) and *LMP* (large molecular weight proteasome) are located in the *HLA-DP* and *-DQ* interval and encode proteins that are involved in antigen processing and presentation. Our earlier work indentified some genomic variants of *TAP* and *LMP* that were associated with chronic hepatitis B and hepatitis C [9],[10].

Among the genes located in the HLA class-II *DQ-DP* interval, eight genes are categorized into antigen processing/presentation-related genes, including *TAP1*, *TAP2*, *LMP2*, *LMP7*, *HLA-DMA*, *HLA-DMB*, *HLA-DOA*, and *HLA-DOB* [11],[12]. *TAP1* and *TAP2* encode the two subunits of TAP. During antigen presentation, TAP works with its binding protein TAPBP, which is encoded by *tapasin* located near the centromere of chromosome 6 [13]. *LMP2* and *LMP7* encode LMP. TAP and LMP transport antigenic peptides from the cytosol into the endoplasmic reticulum in an ATP-dependent manner [14]. *HLA-DMA* and *HLA-DMB* encode two chains of DM complex, which is required for the assembly of antigenic peptides with the HLA class-II molecules [15]. DO, a protein complex translated from *HLA-DOA* and *HLA-DOB*, negatively regulates the activity of DM [16],[17].

Very few studies have investigated the influence of variants in these nine genes on HCV infection [10],[18]. Considering the importance of antigen presentation during immune response to HCV as well as what we have observed previously, we hypothesized that genomic variants of these genes may play a role in generating immune responses and contribute to the outcomes of HCV infection. The current study was to reveal any possible association of these nine genes with HCV in a Chinese population of former paid-blood donors.

## Methods

### Participants

All participants were recruited from a population of former paid-blood donors in Zhenjiang, Jiangsu Province, China. These participants had a high risk of acquiring HCV infection due to improper use of needles and syringes. The study protocol was approved by the institutional review committee of Nanjing Medical University. Interviews for donation history and other risk factors were conducted with signed informed consent from April 2010 to January 2013. An approximately 10 mL of blood sample was then collected for serological tests and host DNA genotyping. Those eligible for the study were excluded if they were positive of any of the following conditions: hepatitis B surface antigen (HBsAg), other types of liver diseases, alcoholic diseases, metabolic liver diseases, and previous interferon and/or ribavirin therapy. The subjects tested to be anti-HCV negative were defined as the uninfected ones. The subjects tested to be anti-HCV positive were defined as the infected cases. The infected cases were further classified into chronic cases (HCV RNA positive) and spontaneous resolvers (HCV RNA negative).

### Laboratory analysis and genotyping of SNPs

Sera HBsAg and HCV antibody (anti-HCV) were detected by enzyme-linked immunosorbent assay (Beijing Wantai Biological Pharmacy Engineering Co., Ltd., Beijing, China) following the manufacturer’s instructions. Blood biochemical tests were undertaken by Roche MODULE P800 Automatic Biochemical Analyzer (Roche Co., Ltd., Shanghai, China). Total RNA was extracted from serum using Trizol LS Reagent (TaKaRa Biotechnology Co., Ltd., Dalian, China), and HCV RNA was detected by RT-PCR with specific primers using RT-PCR kit (TaKaRa Biotechnology Co., Ltd., Dalian, China) [10].

DNA extraction was performed by protease K digestion and phenol-chloroform purification as described previously [10]. The information of single-nucleotide polymorphisms (SNPs) in 9 candidate genes (*HLA-DMA, HLA-DMB, HLA-DOA, HLA-DOB, TAP1, TAP2, LMP2, LMP7,* and *tapasin*) were obtained from the NCBI dbSNP database (http://www.ncbi.nlm.nih.gov/SNP) and the Chinese Han population (CHB) database of HapMap (http://www.hapmap.org). All the SNPs were filtered with the criteria: (1) MAF (minor allele frequency) ≥ 0.05; (2) Hardy-Weinberg equilibrium test *P* value ≥ 0.05. Tag SNPs were selected to represent a group of variants with strong linkage disequilibrium (LD). A total of 34 SNPs were chosen for genotyping. The chosen SNPs were listed in both Additional file 1: Table S1 and S2. Genotyping was performed by the TaqMan allelic discrimination assay on ABI PRISM 7900HT Sequence Detection system (Applied Biosystems, San Diego, CA, USA). The information of primers and probes was shown in Additional file 1: Table S1. Two blank controls and five repeated samples were assigned into each 384-well format for quality control, and a 100% concordance was achieved. The success rates of genotyping for 34 SNPs were all above 95%. The samples failed for genotyping were excluded from the statistical analyses. The observed genotype frequencies of these SNPs in the remaining subjects with different HCV status were all in Hardy-Weinberg equilibrium.

### Statistical analysis

Differences in the general demographic characteristics were calculated by the Student *t* test or One-Way ANOVA and the chi-square (*χ*^2^) test. The associations of SNPs with HCV susceptibility and HCV clearance were estimated by the odds ratios (ORs) and 95% confidence intervals (CIs) using both univariate and multivariate logistic regression analysis. Adjustment for age, gender, history of blood/plasma donation, and times of donation was conducted during regression analysis. The *χ*^2^-based Q test was used for homogeneity among strata by selected variables. The trend analysis was assessed with Cochran-Armitage trend test. LD parameters (i.e., D’ and r^2^) were analyzed by Haploview. The haplotype frequencies based on the observed genotypes were estimated by PHASE software (v2.1) [19],[20]. False discovery rate (FDR) correction was performed as described in reference [21]. All the statistical analyses were carried out by SAS 9.1.3 software (SAS Institute, Cary, NC, USA), and *P* < 0.05 in a two-sided test was considered as statistical significance.

## Results

### Demographic and selected variables of participants

All enrolled participants were divided into three groups based on their anti-HCV and HCV RNA status, including 482 anti-HCV negative subjects (uninfected subjects), 193 spontaneous clearance cases (anti-HCV positive and HCV RNA negative, also called resolvers), and 532 persistent HCV cases (both anti-HCV and HCV RNA positive, also called chronic cases). The later two groups were combined as HCV infected subjects. Some demographic and selected characteristics were shown in Table 1. Distribution of age and gender was not different among groups. Consistent with current knowledge of HCV transmission pattern, experience of plasma donation was associated with HCV infection and the risk of HCV chronicity was increased with the times of plasma donation. The level of aspartate aminotransferase (AST) and alanine aminotransferase (ALT) indicated that HCV infected subjects had worse liver function compared with non-infected subjects.

**Table 1:** **Demographic and selected variables in subjects with different HCV infection outcomes**

Variables	Uninfected	Resolver	Chronic	***P*** value
	(n = 482) N(%)	(n = 193) N(%)	(n = 532) N(%)	
Age, year (mean ± SD)	56.90 ± 8.46	57.32 ± 7.93	57.73 ± 8.05	0.271
Gender				0.054
Male	134 (27.8)	39 (20.2)	120 (22.6)	
Female	348 (72.2)	154 (79.8)	412 (77.4)	
Blood donation				0.770
Never	57 (11.8)	23 (11.9)	56 (10.5)	
Ever	425 (88.2)	170 (88.1)	476 (89.5)	
Numbers of blood donation				0.408
< 30	250 (51.9)	111 (57.5)	282 (53.0)	
≥30	232 (48.1)	82 (42.5)	250 (47.0)	
Plasma donation				<0.001
Never	144 (29.9)	14 (7.3)	29 (5.5)	
Ever	338 (70.1)	179 (92.7)	503 (94.5)	
Numbers of plasma donation				<0.001
<50	363 (75.3)	105 (54.4)	302 (56.8)	
≥50	119 (24.7)	88 (45.6)	230 (43.2)	
ALT (U/L)				<0.001
<40	455 (94.4)	123 (63.7)	324 (60.7)	
≥40	27 (5.6)	70 (36.3)	208 (39.3)	
AST (U/L)				<0.001
<40	435 (90.2)	120 (62.2)	282 (53.0)	
≥40	47 (9.8)	73 (37.8)	250 (47.0)	

*Abbreviation*: *SD* standard deviation.

### Association of candidate SNPs with HCV infection outcomes

Three genetic models (dominant, recessive, and additive models) were used in analysis of each SNP. Significance in any model was considered as a possible association of these SNPs with HCV infection (Additional file 1: Table S2). After adjustment for age, gender, experience of blood/plasma donation, and times of blood/plasma donation, logistic regression analyses showed that variants in *HLA-DMA, HLA-DOA*, *HLA-DOB*, *LMP2*, and *LMP7* were associated with outcomes of HCV infection.

The allelic frequencies of candidate genes were firstly compared among the uninfected controls and infected cases (including both natural clearance cases and chronic cases). *HLA-DMA* rs1063478-T mutant had a decreased frequency in infected cases compared with C wild type. *HLA-DOA* rs2284191-A (*vs.* G) and *HLA-DOB* rs11244-T (*vs.* C) variants were positively related with anti-HCV (Table 2a). Conditional logistic regression analysis was performed to test the independence of these SNPs. The effect of rs11244 was weakened (*P* = 0.078) after being conditioned on the other two SNPs, so it was excluded from further analysis of combined effect (Additional file 1: Table S3a). The combined effect of two independent SNPs (rs1063478-T and rs2284191-G) was analyzed by Cochran-Armitage’s trend test. The results showed an increased protection effect with more favorable alleles (*P* = 0.037). Carrying three favorable alleles offered the highest protective effect (OR = 0.46, 95% CI = 0.27-0.78), as showed in Table 3a.

**Table 2:** **Association of selected SNPs with HCV infection outcomes**

a) SNPs associated with anti-HCV status				
Genotype	Uninfected N(%)	Infected N(%)	OR (95% CI)	***P*** value
rs1063478				
CC	223 (46.3%)	373 (51.4%)	1.00	
CT	216 (44.8%)	297 (41.0%)	0.75 (0.56–0.99)	0.049
TT	43 (8.9%)	55 (7.6%)	0.72 (0.43–1.20)	0.210
Dominant			0.74 (0.56–0.98)	0.035
Recessive			0.83 (0.50–1.35)	0.444
Additive			0.81 (0.65–0.99)	0.049
rs2284191				
GG	395 (82.0%)	572 (78.9%)	1.00	
AG	81 (16.8%)	146 (20.1%)	1.45 (1.01–2.07)	0.430
AA	6 (1.2%)	7 (1.0%)	1.32 (0.32–5.83)	0.701
Dominant			1.44 (1.01–2.05)	0.041
Recessive			1.23 (0.30–5.00)	0.772
Additive			1.38 (1.00–1.92)	0.050
rs11244				
CC	288 (62.4%)	430 (63.1%)	1.00	
CT	147 (31.8%)	211 (21.6%)	1.14 (0.83–1.55)	0.416
TT	27 (5.8%)	40 (5.9%)	1.97 (1.06–3.65)	0.033
Dominant			1.12 (0.84–1.50)	0.426
Recessive			1.74 (0.95–3.18)	0.073
Additive			1.26 (1.00–1.60)	0.053
**b) SNPs associated with HCV chronicity**				
**Genotype**	**Resolver N(%)**	**Chronic N(%)**	**OR (95% CI)**	***P*** **value**
rs17587				
GG	123 (63.7%)	361 (67.9%)	1.00	
AG	58 (30.1%)	158 (29.8%)	0.94 (0.65–136)	0.745
AA	12 (6.2%)	12 (2.3%)	0.32 (0.14–0.75)	0.009
Dominant			0.83 (0.58–1.18)	0.303
Recessive			0.33 (0.14–0.76)	0.009
Additive			0.76 (0.57–1.03)	0.077
rs2071543				
CC	125 (64.7%)	356 (66.9%)	1.00	
AC	54 (28.0%)	157 (29.5%)	1.06 (0.73–1.55)	0.756
AA	14 (7.3%)	19 (3.6%)	0.49 (0.24–1.02)	0.058
Dominant			0.94 (0.66–1.34)	0.749
Recessive			0.48 (0.24–0.99)	0.049
Additive			0.86 (0.65–1.15)	0.318
rs2284191				
GG	142 (73.6%)	430 (80.3%)	1.00	
AG	50 (25.9%)	96 (18.1%)	0.59 (0.40, 0.88)	0.010
AA	1 (0.5%)	6 (1.1%)	1.41 (0.15, 12.89)	0.760
**Genotype**	**Resolver N(%)**	**Chronic N(%)**	**OR (95% CI)**	***P*** **value**
Dominant			0.61 (0.41, 0.90)	0.014
Recessive			1.59 (0.17, 14.43)	0.683
Additive			0.65 (0.45, 0.95)	0.240
rs376892				
CC	109 (56.5%)	321 (60.3%)	1.00	
CT	81 (42.0%)	183 (34.4%)	0.67 (0.54–1.07)	0.112
TT	3 (1.5%)	28 (5.3%)	3.15 (0.93–10.64)	0.065
Dominant			0.84 (0.60–1.18)	0.317
Recessive			3.51 (1.04–11.77)	0.042
Additive			0.98 (0.74–1.32)	0.918
rs416622				
GG	66 (34.2%)	242 (45.5%)	1.00	
AG	105 (54.4%)	216 (40.6%)	0.51 (0.35–0.74)	<0.001
AA	22 (11.4%)	74 (13.9%)	0.91 (0.52–1.59)	0.739
Dominant			0.58 (0.41–0.83)	0.003
Recessive			1.31 (0.78–2.19)	0.300
Additive			0.82 (0.64–1.04)	0.102
rs7383287				
AA	125 (64.8%)	391 (73.5%)	1.00	
AG	25 (13.0%)	67 (12.6%)	0.84 (0.50–1.39)	0.495
GG	43 (22.2%)	74 (13.9%)	0.54 (0.35–0.85)	0.008
Dominant			0.66 (0.46–0.95)	0.025
Recessive			0.56 (0.36–0.87)	0.001
Additive			0.75 (0.60–0.93)	0.001

Logistic regression analyses adjusted for age, gender, experience of blood/plasma donation, and numbers of blood/plasma donation.

**Table 3:** **Cumulative effects of selected SNPs on HCV infection outcomes**

a) Combined favorable alleles (rs1063478–T and rs2284191–G) and anti–HCV			
Variables	Uninfected N(%)	Infected N(%)	OR (95% CI)
0–1	37 (7.7)	75 (10.0)	1.00
2	227 (47.1)	364 (48.4)	0.62 (0.37–1.03)
3	188 (39.0)	245 (32.6)	0.46 (0.27–0.78)
4	30 (6.2)	41 (9.0)	0.50 (0.24–1.04)
Trend			*P* ^a^ = 0.037
0–2	264 (54.8)	439 (58.4)	1.00
3–4	218 (45.2)	286 (41.6)	0.70 (0.53–0.93)
**b) Combined favorable alleles (rs17587–A and rs7383287–G) and HCV chronicity**			
**Variables**	**Resolver N(%)**	**Chronic N(%)**	**OR (95% CI)**
0	71 (36.8)	257 (48.3)	1.00
1	62 (32.1)	177 (33.3)	0.79 (0.53–1.77)
2	50 (25.9)	74 (13.9)	0.42 (0.26–0.66)
3–4	10 (5.2)	23 (4.5)	0.62 (0.27–1.37)
Trend			*P* ^a^ = 0.001
0	71 (36.8)	257 (48.3)	1.00
1–4	122 (63.2)	275 (51.7)	0.63 (0.45–0.88)

Logistic regression analyses adjusted for age, gender, experience of blood/plasma donation, and numbers of blood/plasma donation.

^a^ *P* value of Cochran-Armitage’s trend test.

The ability of viral clearance was then compared among the spontaneous infection subjects and the persistent infection subjects. *LMP2* rs17587-A (*vs.* G), *LMP7* rs2071543-A (*vs.* C), *HLA-DOA* rs2284191-A (*vs.* G), rs376892-T (*vs.* C), rs416622-A (*vs.* G), and *HLA-DOB* rs7383287-G (*vs.* A) were positively associated with HCV clearance (Table 2b). The effect of rs17587 and rs7383287 on HCV clearance remained obvious after being conditioned on the other five SNPs (*P* = 0.03 for rs17587 and *P* = 0.007 for rs7383287) (Additional file 1: Table S3b). Therefore, the independent SNPs of *LMP2* rs17587 and *HLA-DOB* rs7383287 were analyzed in Cochran-Armitage’s trend test. There was also an increased protection effect with more favorable alleles (*P* = 0.001). Subjects carrying two favorable alleles had a 58% decrease in risk of HCV persistent infection (OR = 0.42, 95% CI = 0.26-0.66) (Table 3b).

### Stratified analysis of independent SNPs

The association between the combined effect of the independent SNPs and HCV infection was further evaluated by adjustment for confounding factors including age, gender, experience of blood/plasma donation, and times of blood/plasma donation. The results were shown in Additional file 1: Table S4.

The combined protective effect of *HLA-DMA* rs1063478-T and *HLA-DOA* rs2284191-G was more prominent in female subgroup (OR = 0.68, 95% CI = 0.49-0.94). Subjects < 60 years favored more from the protective effect of rs1063478-T and rs2284191-G as compared with subjects ≥ 60 years (OR = 0.62, 95% CI = 0.43-0.88). The protective effect of carrying rs1063478-T and rs2284191-G was also more obvious in subjects with experience of blood donation (OR = 0.69, 95% CI = 0.51-0.94), subjects with blood donation ≥ 30 times (OR = 0.61, 95% CI = 0.40-0.93), subjects with experience of plasma donation (OR = 0.66, 95% CI = 0.49-0.90), and subjects with plasma donation ≥ 50 times (OR = 0.67, 95% CI = 0.47-0.95). Heterogeneity test showed that heterogeneity in every two strata was significant for gender and plasma donation (*P* = 0.023 and 0.024, respectively).

The combined protective effect of *LMP2* rs17587-A and *HLA-DOB* rs7383287-G was more pronounced in female subjects (OR = 0.63, 95% CI = 0.43-0.93), subjects < 60 years (OR = 0.49, 95% CI = 0.31-0.78), subjects with experience of blood donation (OR = 0.67, 95% CI = 0.47-0.97), subjects with blood donation < 30 times (OR = 0.50, 95% CI = 0.31-0.81), subjects with experience of plasma donation (ever *vs.* never, OR = 0.61, 95% CI = 0.43-0.87), and subjects with plasma donation ≥ 50 times (OR = 0.46, 95% CI = 0.27-0.78). No obvious evidence of heterogeneity associations was observed.

### Interaction analysis

The interaction between the meaningful SNPs and potential risk factors was also analyzed. The results were shown in Table 4. Significant multiplicative interactions on HCV susceptibility were found between the combined effects of rs1063478 and rs2284191 and plasma donation (*P*_interaction_ = 0.020). Compared to subjects carrying 0-2 favorable alleles and with experience of plasma donation, subjects carrying 3-4 favorable alleles but without plasma donation had a decreased detection rate of anti-HCV (OR = 0.18, 95% CI = 0.10-0.32). There was also a multiplicative interaction between rs7383287 genotypes and exposure of blood donation (*P*_interaction_ = 0.040). Compared to subjects carrying rs7383287AA genotypes and with experience of blood donation, subjects carrying AG/AA genotypes but without blood donation had a 66% decrease in risk of HCV persistent infection (OR = 0.34, 95% CI = 0.15-0.78).

**Table 4:** **Interaction analysis between favorable SNPs and selected risk factors**

a) Combined favorable alleles (rs1063478-T and rs2284191-G) and history of plasma donation			
Variables	Uninfected N(%)	Infected N(%)	OR (95% CI)
0-2 favorable alleles with plasma donation	184 (40.7)	421 (58.1)	1.00
3-4 favorable alleles with plasma donation	154 (34.1)	261 (36.0)	0.66 (0.49-0.89)
0-2 favorable alleles without plasma donation	80 (17.7)	18 (2.5)	0.16 (0.08-0.30)
3-4 favorable alleles without plasma donation	64 (7.5)	25 (3.4)	0.18 (0.10-0.32)
*P* for multiplicative interaction			*P* = 0.020
**b) rs7383287 genotypes and history of blood donation**			
**Variables**	**Resolver N(%)**	**Chronic N(%)**	**OR (95% CI)**
AA genotypes with blood donation	115 (59.6)	349 (65.6)	1.00
AG/GG genotypes with blood donation	10 (5.2)	42 (7.9)	1.39 (0.64-2.99)
AA genotypes without blood donation	55 (28.5)	127 (23.9)	0.76 (0.51-1.13)
AG/GG genotypes without blood donation	13 (6.7)	14 (2.6)	0.34 (0.15-0.78)
*P* for multiplicative interaction			*P* = 0.040

Logistic regression analyses adjusted for age, gender, experience of blood/plasma donation, and numbers of blood/plasma donation.

### Haplotype analysis

Because high linkage disequilibrium exists in HLA region, we also analyzed the LD among the candidate SNPs (detailed information showed in Additional file 1: Table S5) and then we performed haplotype analysis.

In the above analyses, rs1063478, rs2284191, and rs11244 were identified to be related with anti-HCV status. Compared with the most frequent CGC haplotype, the haplotype with rs1063478-T (TGC) was associated with a protective effect (*P* < 0.001), while the haplotype carrying rs2284191-A (CAC) indicated a risk effect of HCV infection (*P* < 0.001) (Table 5a).

**Table 5:** **Haplotype analysis with different HCV outcomes**

a) Haplotype analysis of rs1063478, rs2284191, and rs11244 in sequence				
Haplotype	Uninfected N(%)	Infected N(%)	OR (95% CI)	***P***
CGC	461 (47.8)	719 (49.6)	1.00	–
TGC	232 (24.1)	290 (20.0)	0.58 (0.47–0.73)	<0.001
CGT	161 (16.7)	241 (16.6)	1.22 (0.94–1.58)	0.125
TAC	53 (5.5)	76 (5.2)	0.79 (0.53–1.17)	0.232
CAC	17 (1.8)	74 (5.1)	3.35 (1.87–5.99)	<0.001
Others^*^	40 (4.1)	50 (3.5)	1.15 (0.71–1.87)	0.572
**b) Haplotype analysis of rs17587, rs2071543, rs2284191, rs7383287, rs376892, and rs416622 in sequence**				
**Haplotype**	**Resolver N(%)**	**Chronic N(%)**	**OR (95% CI)**	***P***
GCGACG	101 (26.2)	213 (20.0)	1.00	–
GCGGCG	36 (9.3)	18 (1.7)	0.24 (0.13–0.45)	<0.001
ACGACG	34 (8.8)	26 (2.4)	0.37 (0.21–0.65)	0.001
GAGACG	33 (8.5)	73 (6.9)	1.06 (0.66–1.71)	0.818
GCGGTA	24 (6.2)	38 (3.6)	2.37 (1.42–3.94)	0.001
GCGATA	13 (3.4)	89 (8.4)	3.31 (1.75–6.25)	<0.001
GAGGCG	13 (3.4)	59 (5.5)	2.09 (1.09–4.02)	0.027
Others^*^	386 (34.2)	566 (51.5)	1.76 (1.28–2.41)	<0.001

Logistic regression analyses adjusted for age, gender, experience of blood/plasma donation, and numbers of blood/plasma donation.

^*^Haplotypes with a frequency less than 5% in both groups were combined as others.

Six SNPs were identified to be related with chronicity of HCV infection, including rs17587, rs2071543, rs2284191, rs7383287, rs376892 and rs416622. The most frequent haplotype was GCGACG. No difference of viral clearance was found between reference haplotype GCGACG and haplotype carrying rs2071543 (GAGACG). Haplotypes carrying rs7383287-G (GCGGCG) and rs17587-A (ACGACG) were more frequently found in spontaneous clearance group than in chronic infection group (*P* < 0.001 and *P* = 0.001, respectively). Haplotypes carrying the other alleles enhanced the risk of chronic HCV infection. The results were shown in Table 5b.

## Discussion

HCV infection is now considered as curable and therefore lots of studies including several GWAS have focused on genetic polymorphisms and treatment response [22],[23]. Comparatively fewer data reported association of genetic variants with HCV infection outcomes. A recent GWAS study demonstrated that SNPs near *interleukin-28B* (*IL-28B*) and *DQB* might explain approximately 15% of spontaneous resolution of HCV infection with European and African ancestry [24]. This GWAS together with other studies have revealed that HLA is one of the most important regions with respect to viral hepatitis infection. However, there are still some shortages of relative studies about HCV infection outcomes. One major difficulty is owing to sample collection because HCV prevalence in general population is very low. A detailed review compared studies from different ethnic groups and speculated that the chance was limited to detect globally common HLA haplotypes due to high diversity in HLA loci [25]. Current GWAS data are based on mixed populations and most published high quality studies were conducted in non-Chinese population. Conventional genotyping for precise detection of HLA loci in specific population is definitely necessary to complement GWAS approach. The present study investigated a HLA region with little attention and defined several new genomic loci that were related with HCV susceptibility and clearance in Chinese Han population. We selected and analyzed 34 tagging-SNPs in 9 candidate genes (*HLA-DMA, HLA-DMB, HLA-DOA, HLA-DOB, TAP1, TAP2, LMP2, LMP7*, and *tapasin*), which are involved in antigen processing and presentation. The results indicated that *HLA-DMA* rs1063478 and *HLA-DOA* rs2284191 were independent factor of being anti-HCV positive, while *HLA-DOB* rs7383287 and *LMP2* rs17587 were independent factor of infection chronicity. Stratified analyses and haplotype analyses all showed that rs1063478-T mutant and rs2284191-G wild type had protective effect from HCV infection, and wild type rs17587-A with rs7383287-G could help clear virus.

Variants in *HLA-DMA*, *HLA-DOA,* and *HLA-DOB* were reported for the first time to play a role in HCV infection. *HLA-DMA* rs1063478 C > T makes a missense mutation. Mutation may change the activity of the encoding protein and influence antigen presentation process. *HLA-DOA* rs2284191 (G > A) was in intron region. *HLA-DOB* rs7383287 (A > G) make a synonymous mutation. It is hard to explain the biological plausibility of those two SNPs without further studies. However, *HLA-DOB* rs7383287 seems to be a strong biomarker of HCV clearance, because the association was significant in dominant, recessive, and additive all three models (Table 2b).

*LMP2* rs17587 G > A also makes a missense mutation. Based on our previous study conducted in injecting drug users, *LMP2* rs17587 had no relationship with the outcomes of HCV infection, which was not in accordance with this study [10]. Another study in a European Caucasian population found that G allele in exon 4 of *tapasin* was associated with outcomes of HCV infection, which was not observed in out study [18]. The discrepancy among these studies may be due to the different study design as well as the participants with dissimilar physical conditions and genetic background. The inconsistency among studies also illustrates that collecting data from specific population should help reveal unique disease-related effects in a given genetic background rather than in generalized population setting.

Some methodological issues need to be mentioned. Firstly, selection bias may be induced during sample collection. Although only subjects with risk exposure of paid-blood and/or plasma donation were recruited, some uninfected controls may never have contact with the pathogen. Therefore the uninfected status may not be owned to carrying protective alleles and the estimated risk association would be inaccurate. Nevertheless, considering the frequency and lasting period of blood donation, most subjects should have exposure history and this bias might be minimal. The selected population also has other advantages. The subjects were exposed during the same period and their infection outcomes were steady after decade. The subjects live in the same area and share similar environment exposure. All the advantages make the current population one of the best choice for studying the natural history of HCV infection. Secondly, 34 SNPs were included in the present study, which may increase the false positive results because of multiple comparisons. Therefore the association found in this study could be a false-positive result. For this reason we performed FDR correction for all SNPs, which was provided in Additional file 1: Table S2. As a result, *HLA-DOB* rs7383287 and *LMP2* rs17587 were highly possible to be related with HCV chronicity because the FDR was pretty small in all three genetic models (FDR < 0.3 as significance). Although we analyzed 34 SNPs, other SNPs covering more regions might also contribute to the risk of HCV chronicity. It should be noticed that the association strength of *HLA-DOB* rs7383287 and *LMP2* rs17587 could be weakened in a larger scale study as GWAS. Frankly speaking, future work with more subjects and covering more SNPs is needed to confirm this association as well as to explore other potential loci. Finally, HCV viral genotype may be a confounding factor and we did not include in analysis. Based on our current knowledge, HCV 1b strain is the dominant strain in China and accounts for over half of the HCV cases we collected [9],[10],[26],[27]. We did not adjust viral genotype in analysis due to insufficient subgroup sample size. More samples are needed for further stratified analysis.

## Conclusion

Our results suggested that genetic variants in antigen presentation pathway had influence on susceptibility to HCV infection and viral clearance. *HLA-DMA* rs1063478, *HLA-DOA* rs2284191, and *HLA-DOB* rs7383287 were identified as novel loci in Chinese population that were involved in HCV infection.

## Additional file

## Electronic supplementary material

Additional file 1: **Table S1.** gave information of primers and probes for TaqMan allelic discrimination. **Table S2.** showed the results of SNPs distribution using dominant, recessive, or additive models. **Table S3.** showed the results of conditional analyses of selected SNPs in additive genetic model. **Table S4.** showed the results of stratified analyses of combined favorable alleles and HCV infection outcomes. **Table S5.** gave linkage disequilibrium information in uninfected subjects. (PDF 337 KB)

## Abbreviations

HCV: Hepatitis C virus
HLA: Human leukocyte antigen
TAP: Transporter associated with antigen processing
LMP: Large molecular weight proteasome
HBsAg: Hepatitis B surface antigen
SNP: Single-nucleotide polymorphisms
CHB: Chinese Han population
MAF: Minor allele frequency
LD: Linkage disequilibrium
OR: Odds ratio
CI: Confidence interval
FDR: False discovery rate
AST: Aspartate aminotransferase
ALT: Alanine aminotransferase
GWAS: Genome-wide association studies

## References

[1] Lavanchy D (2011). Evolving epidemiology of hepatitis C virus. Clin Microbiol Infect.

[2] Seto WK, Lai CL, Fung J, Hung I, Yuen J, Young J, Wong DKH, Yuen MF (2010). Natural history of chronic hepatitis C: Genotype 1 versus genotype 6. J Hepatol.

[3] Halfon P, Locarnini S (2011). Hepatitis C virus resistance to protease inhibitors. J Hepatol.

[4] Ghany MG, Strader DB, Thomas DL, Seeff LB (2009). Diagnosis, management, and treatment of hepatitis C: An update. Hepatology.

[5] Fernando MMA, Stevens CR, Walsh EC, De Jager PL, Goyette P, Plenge RM, Vyse TJ, Rioux JD (2008). Defining the role of the MHC in autoimmunity: a review and pooled analysis. PLoS Genet.

[6] Rauch A, Gaudieri S, Thio C, Bochud PY (2009). Host genetic determinants of spontaneous hepatitis C clearance. Pharmacogenomics.

[7] Tamori A, Kawada N (2013). HLA class II associated with outcomes of hepatitis B and C infections. World J Gastroenterol.

[8] Yu RB, Hong X, Ding WL, Tan YF, Zhang YX, Sun NX, Wu GL, Zhan SW, Ge DF (2008). The association between the genetic polymorphism of HLA-DQA1, DQB1, and DRB1 and serum alanine aminotransferase levels in chronic hepatitis C in the Chinese population. J Gastroen Hepatol.

[9] Shi C, Qian YH, Su J, Luo SS, Gu J, You H, Cui Q, Lin YD, Dong MH, Yu RB (2011). Genetic variation in the LMP/TAP gene and outcomes of hepatitis B virus infection in the Chinese population. Epidemiol Infect.

[10] Cui Q, Zhang Y, Su J, Shi C, Lei N, Ding K, Li J, Yu R, Wang L, Wang N (2010). The association between the genetic polymorphisms of LMP2/LMP7 and the outcomes of HCV infection among drug users. J Biomed Res.

[11] Horton R, Wilming L, Rand V, Lovering RC, Bruford EA, Khodiyar VK, Lush MJ, Povey S, Talbot CC, Wright MW (2004). Gene map of the extended human MHC. Nat Rev Genet.

[12] Shiina T, Hosomichi K, Inoko H, Kulski JK (2009). The HLA genomic loci map: expression, interaction, diversity and disease. J Hum Genet.

[13] Peh CA, Laham N, Burrows SR, Zhu Y, McCluskey J (2000). Distinct functions of Tapasin revealed by polymorphism in MHC class I peptide loading. J Immunol.

[14] Romieu-Mourez R, François M, Boivin MN, Stagg J, Galipeau J (2007). Regulation of MHC class II expression and antigen processing in murine and human mesenchymal stromal cells by IFN-γ, TGF-β, and cell density. J Immunol.

[15] Anders AK, Call MJ, Schulze MSED, Fowler KD, Schubert DA, Seth NP, Sundberg EJ, Wucherpfennig KW (2011). HLA-DM captures partially empty HLA-DR molecules for catalyzed removal of peptide. Nat Immunol.

[16] Neefjes J, Jongsma MLM, Paul P, Bakke O (2011). Towards a systems understanding of MHC class I and MHC class II antigen presentation. Nat Rev Immunol.

[17] Denzin LK, Fallas JL, Prendes M, Yi W (2005). Right place, right time, right peptide: DO keeps DM focused. Immunol Rev.

[18] Ashraf S, Nitschke K, Warshow UM, Brooks CR, Kim AY, Lauer GM, Hydes TJ, Cramp ME, Alexander G, Little AM, Thimme R, Neumann-Haefelin C, Khakoo SI (2013). Synergism of tapasin and HLA in resolving HCV infection. Hepatology.

[19] Stephens M, Donnelly P (2003). A comparison of bayesian methods for haplotype reconstruction from population genotype data. Am J Hum Genet.

[20] Stephens M, Smith NJ, Donnelly P (2001). A new statistical method for haplotype reconstruction from population data. Am J Hum Genet.

[21] Benjamini Y, Hochberg Y (1995). Controlling the false discovery rate: A practical and powerful approach to multiple testing. J Roy Statis Soc Ser B.

[22] Rauch A, Rohrbach J, Bochud PY (2010). The recent breakthroughs in the understanding of host genomics in hepatitis C. Eur J Clin Invest.

[23] Tanaka Y, Nishida N, Sugiyama M, Tokunaga K, Mizokami M (2010). λ-Interferons and the single nucleotide polymorphisms: A milestone to tailor-made therapy for chronic hepatitis C. Hepatol Res.

[24] Priya D, Thio CL, Wojcik GL, Goedert JJ, Mangia A, Latanich R, Kim AY, Lauer GM, Chung RT, Peters MG, Kirk GD, Metha SH, Cox AL, Khakoo SI, Alric L, Cramp ME, Donfield SM, Edlin BR, Tobler LH, Busch MP, Alexander G, Rosen HR, Gao X, Abdel-Hamid M, Apps R, Carrington M, Thomas DL (2013). Genome-wide association study of spontaneous resolution of hepatitis C virus infection: Data from multiple cohorts. Ann Intern Med.

[25] Singh R, Kaul R, Kaul A, Khan K (2007). A comparative review of HLA associations with hepatitis B and C viral infections across global populations. World J Gastroenterol.

[26] Bacon BR, Gordon SC, Lawitz E, Marcellin P, Vierling JM, Zeuzem S, Poordad F, Goodman ZD, Sings HL, Boparai N, Burroughs M, Brass CA, Albrecht JK, Esteban R (2011). Boceprevir for previously treated chronic HCV genotype 1 infection. N Engl J Med.

[27] Poordad F, McCone J, Bacon BR, Bruno S, Manns MP, Sulkowski MS, Jacobson IM, Reddy KR, Goodman ZD, Boparai N, DiNubile MJ, Sniukiene V, Brass CA, Albrecht JK, Bronowicki JP (2011). Boceprevir for untreated chronic HCV genotype 1 infection. N Engl J Med.

