# Supplementary material for: Genetic variants in antigen presentation-related genes influence susceptibility to hepatitis C virus and viral clearance: a case control study
Source: BMC Infect Dis. 2014 Dec 21;14:716. doi: 10.1186/s12879-014-0716-8 (PMC4279674; doi:10.1186/s12879-014-0716-8)
Supplement: Supplementary file 1 — Additional file 1: Table S1. gave information of primers and probes for TaqMan allelic discrimination. Table S2. showed the results of SNPs distribution using dominant, recessive, or additive models. Table S3. showed the results of conditional analyses of selected SNPs in additive genetic model. Table S4. showed the results of stratified analyses of combined favorable alleles and HCV infection outcomes. Table S5. gave linkage disequilibrium information in uninfected subjects. (PDF 337 KB) [file 12879_2014_716_MOESM1_ESM.pdf]

Supplemental Table 1. Information of primers and probes for TaqMan allelic discrimination

Polymorphism		Sequence(5'-3')
DMA rs1050391	Primer	F: AGGTCTTCTTCCAGGCAAGGA<br>R: GAGCTATAGACAGGAAGTGCTGAA
	Probe	FAM-CTAGACGTAGAAGCAG-MGB<br>HEX-AGCTAGACATAGAAGCAGA-MGB
DMA rs1063478	Primer	F: CCACCCATGCTGACAGTGA<br>R: AGTCCATCGACAGCTGAGACA
	Probe	FAM-ATCATTCCGTCCCTGTG-MGB<br>HEX-CATCATTCCATCCCTGT-MGB
DMA rs3135029	Primer	F: TCGCTTGGGTGTTCCTGTATAA<br>R: CAGGGCCCACGTGATCTG
	Probe	FAM-TAAGCTACAAATTCTGGCA-MGB<br>HEX-ATAAGCTACAAATTCGGGCAA-MGB
DMB rs23544	Primer	F: CCACTGTATTAGAGAGGACCTGGAA<br>R: ACCACCCACTCCCAAGAAAAT
	Probe	FAM-AGATGGATTCCCC-MGB<br>HEX-AGATGGGTTCCCC-MGB
DMB rs2071556	Primer	F: GTTGAAATCTAAGGAGGCTAGAATAGTGTA<br>R: GGAGCGTGCTTGCTTTGAA
	Probe	FAM-ATTGCCAAATTTACAGCC-MGB<br>HEX-TGCCAAATTTACATCCT-MGB
DOA rs408036	Primer	F: CCAGGCCTTGGCCAGTT<br>R: GTAACACACAATGGGCCAAATG
	Probe	FAM-TTGGCAGCCGTCCT-MGB<br>HEX-ATTGGCAGCCATC-MGB
DOA rs3128935	Primer	F: TGTCGGGTGGACATGTTCAC<br>R: GGATCCACATGGTCTGTGTTCTC
	Probe	FAM-AGAACACCGCTAACA-MGB<br>HEX-AGAACACCGCCAACA-MGB
DOA rs3129304	Primer	F: AAAACATACAAAGAGATAAATCACCATACC<br>R: TGAAAACCGTAATCTGTATTGCTCAT
	Probe	FAM-CATAGTTTATGTCAGGACC-MGB<br>HEX-CATAGTTTATGTCAAGACC-MGB
DOA rs376892	Primer	F: CTTGGCTGTGGTCTGGTAACTG<br>R: CCTTCCTAGTCCACCTCAGACCTT
	Probe	FAM-TAATCAGGTGCCATTGG-MGB<br>HEX-TAATCAGGTGCCATCGG-MGB
DOA rs369150	Primer	F: GAAAGAAAGGAACAGGGCATGAC<br>R: GGCGGGAAGGTCCAGAGA
	Probe	FAM-TGATGGGAACCTAGG-MGB<br>HEX-TGATGGGAGCCTAGG-MGB
DOA rs86567	Primer	F: GGTGCGGGTCTACAGATGGTT<br>R: GAGCAACAGTTATTGAGGAACTAGCAT
	Probe	FAM-TGGCCCCCCATTG-MGB<br>HEX-TGGCCCACCATTG-MGB
DOA rs2284191	Primer	F: TCCTCCATCTCAGAGCATTATGAC<br>R: TGTTGCTCAAACAACTTCATAGAGTTC
	Probe	FAM-CTTCCATAACTGTTGTCTAG-MGB<br>HEX-TAACTGTTATCTAGTTTTCTGG-MGB
DOA rs6913008	Primer	F: GTCCTGTTCAGAGTCATCCACTTT<br>R: TCCTCATCATCATGGGCACAT

Polymorphism		Sequence(5’-3’)
DOA rs6913008	Probe	FAM-CCCAGACTCCCGG-MGB<br>HEX-CCCAGACTCCTGG-MGB
DOA rs2582	Primer	F: TGATCCTTCTGAGAGAAATGACTTGT<br>R: CACAGCGGGATGCACTTAAA
	Probe	FAM-TGTGACAGACCCTGC-MGB<br>HEX-TGTGACAGCCCCTG-MGB
DOA rs416622	Primer	F: CAGCCTGGTGACAGAGTGAGA<br>R: TCACCCAGACCTACTGAATTAGAATCT
	Probe	FAM-AGACAGCCCCCCTGT-MGB<br>HEX-AGACAGCCTCCCTGTT-MGB
DOA rs453779	Primer	F: GTCACCCGTGGAGGCACTA<br>R: AACGTCCCTTAATCCCAGTCCTA
	Probe	FAM-AGGAACAGGCCCTG-MGB<br>HEX-AGGAACGGGCCCTG-MGB
DOA rs1044429	Primer	F: TCACACAAAGAGGGTTTCTGTTACTG<br>R: GAATAAGTTGAAATCAATGACCAGAAGA
	Probe	FAM-TGAGATGATTCTCCTCCAC-MGB<br>HEX-TGAGATGATTTTCCTCCAC-MGB
DOB rs2857111	Primer	F: TCTCTTGCCTCCGTTCTCATTC<br>R: TGCTACATATTTCTAAAAGCCACTCTCATA
	Probe	FAM-TCCCCTCCCTGGAGA-MGB<br>HEX-CTCCCCTCCCTAGAG-MGB
DOB rs11244	Primer	F: CAAGGGCATTGTCCAGAAACTC<br>R: GCTGGACCACAGAAAAGTAAATGA
	Probe	FAM-ACTCCTTCCAGTCCCA-MGB<br>HEX-CTCCTTCCAGCCCCA-MGB
DOB rs1383258	Primer	F: TTACCAGACACGTTTAGAATGGATTC<br>R: GAGTTCACAGCACATTGTAATTATTGG
	Probe	FAM-AGAAGAGATGAGAGAGTC-MGB<br>HEX-CAAGAGAAGAGACGAGAG-MGB
DOB rs2071469	Primer	F: GAAGCCAGAAAAACAGCGATTTAT<br>R: AGGGGACCCACCCAGAAC
	Probe	FAM-TGAATTTTATGAACTCATTC-MGB<br>HEX-TGAATTTTATGAGCTCATTC-MGB
DOB rs2071472	Primer	F: GACTGGATTCCTCCATGACTCAA<br>R: CATGCCAATTCTTGCATACACA
	Probe	FAM-AACAGAGCAATTGTT-MGB<br>HEX-AACAGAGCAATTATT-MGB
DOB rs7383287	Primer	F: CGTAATTTACCAGGCATGGGTTT<br>R: CAGTCAGCCTTTGCCTGAATC
	Probe	FAM-TTCCAGAAGATTTTG-MGB<br>HEX-TTTCCAGAAGACTTTG-MGB
DOB rs2856997	Primer	F: CCAAATCCAATGCTAGCTAGAGAAA<br>R: ATGGGCTGTGAGAATCTGTAACC
	Probe	FAM-CATGGAGTTACCCCC-MGB<br>HEX-CCATGGAGTTACCACC-MGB
DOB rs2071471	Primer	F: CATGCCAATTCTTGCATACACACT<br>R: GGTGGCTCTGCTAGTGAATCTGA
	Probe	FAM-CTGGAGAATCTGTGC-MGB<br>HEX-CTGGAGAGTCTGTGC-MGB
DOB rs2071475	Primer	F: GGTCCTCTCTGGGTACACTGTCA<br>R: GGTTTTCTTTCACGGTGTCTCAT
	Probe	FAM-CTAGGAAGGGAGGAAA-MGB<br>HEX-ACTAGGAAGAGAGGAAA-MGB

Polymorphism		Sequence(5'-3')
DOB rs2856995	Primer	F: CTCCCCTCCCTAGAGAATCCA<br>R: AAGAAACAGAATGGGATTCAACAGA
	Probe	FAM-TGTCTGGTATGTCTCTAA-MGB<br>HEX-TGTCTGGTAAGTCTC-MGB
TAP1 rs1135216	Primer	F: CACACATGTGGCTATACCGTTCTC<br>R: TCGCTGACCCCCTGACA
	Probe	FAM-TGCAGAGGTAGGCG-MGB<br>HEX-TCTGCAGAGGTAGACG-MGB
TAP2 rs1800454	Primer	F: CCTGGAACGCGCCTTGTA<br>R: CCTTTCACAACCACTCTGGTATCTT
	Probe	FAM-TGCTCGTAAGGAGG-MGB<br>HEX-CTGCTCATAAGGAGG-MGB
LMP2 rs17587	Primer	F: TGAACCGAGTGTTTGACAAGCT<br>R: GCTGAACCAGAGAGTGCACAGT
	Probe	FAM- CTGCACGAGCGCA-MGB<br>HEX- CTGCACGAGCACAT-MGB
LMP7 rs2071543	Primer	F: GCGACCCTCCACTCCTCA<br>R: GGACACTACAGTTTCTCTATGCGATCT
	Probe	FAM- CCGACCTTCATTCC-MGB<br>HEX- CCGACCTGCATTC-MGB
tapasin rs9277972	Primer	F: GTCTAGGTCCTTCAGGTAGAAGTAATCTTT<br>R: CTAAGTGAAATTGCATACTGTTTTTACTCTAC
	Probe	FAM-CCTATAAGGTTAAACTGTTCT-MGB<br>HEX-CCTATAAGGTTTAACTGTTCT-MGB
tapasin rs1059288	Primer	F: TGGGCCTTAGGTCCCTATGC<br>R: AAGTGATCGTGTGAGTCGTCGTT
	Probe	FAM-CAGACAGGCCGGTC-MGB<br>HEX-ACAGACAGGCCAGTC-MGB
tapasin rs2282851	Primer	F: CCTCATTCTTGAATTATCTGCACAGT<br>R: GCCCAGGAGTCAGAAGCTTTT
	Probe	FAM-CCACGTCTCAGCCTA-MGB<br>HEX-CCACGTCCCAGCCT-MGB

Supplemental Table 2. Comparison results of SNPs distribution in dominant, recessive, and additive models

SNPs	Location	Dominant		Recessive		Additive		Dominant		Recessive		Additive	
		P Value*	FDR*	*P* Value*	FDR*	*P* Value*	FDR*	*P* Value†	FDR†	*P* Value†	FDR†	*P* Value†	FDR†
DMA rs1050391	intron(C>T)	1.25×10^{-1}	0.64	7.70×10^{-1}	0.85	1.60×10^{-1}	0.68	4.66×10^{-1}	0.79	3.54×10^{-1}	0.75	3.47×10^{-1}	0.84
DMA rs1063478	Missense(C>T)	3.50×10^{-2}	0.64	4.44×10^{-1}	0.82	4.90×10^{-2}	0.68	8.47×10^{-1}	0.96	6.81×10^{-1}	0.88	7.44×10^{-1}	0.90
DMA rs3135029	5'near gene(C>A)	1.66×10^{-1}	0.64	7.43×10^{-2}	0.82	1.95×10^{-1}	0.71	3.43×10^{-1}	0.73	6.95×10^{-1}	0.88	3.44×10^{-1}	0.84
DMB rs23544	intron(G>A)	7.50×10^{-2}	0.64	2.88×10^{-1}	0.82	6.60×10^{-2}	0.68	1.83×10^{-1}	0.72	3.56×10^{-1}	0.75	5.71×10^{-1}	0.87
DMB rs2071556	intron(C>A)	3.32×10^{-1}	0.74	2.66×10^{-1}	0.82	2.08×10^{-1}	0.71	6.95×10^{-1}	0.91	3.49×10^{-1}	0.75	4.48×10^{-1}	0.87
DOA rs408036	3'UTR(G>A)	3.14×10^{-1}	0.74	7.18×10^{-1}	0.85	6.64×10^{-1}	0.88	7.21×10^{-1}	0.91	1.90×10^{-1}	0.75	5.92×10^{-1}	0.87
DOA rs3128935	3'UTR(T>C)	3.11×10^{-1}	0.74	5.05×10^{-1}	0.82	2.98×10^{-1}	0.72	2.18×10^{-1}	0.72	3.76×10^{-1}	0.75	1.75×10^{-1}	0.82
DOA rs3129304	3'UTR(A>G)	5.12×10^{-1}	0.83	2.16×10^{-1}	0.82	3.85×10^{-1}	0.86	6.50×10^{-1}	0.91	9.90×10^{-1}	0.99	6.66×10^{-1}	0.87
DOA rs376892	3'UTR(C>T)	5.43×10^{-1}	0.83	8.30×10^{-2}	0.82	2.55×10^{-1}	0.72	3.17×10^{-1}	0.72	4.20×10^{-2}	0.42	9.18×10^{-1}	0.92
DOA rs369150	intron(G>A)	5.64×10^{-1}	0.83	9.98×10^{-1}	1.00	6.85×10^{-1}	0.88	3.00×10^{-1}	0.72	3.29×10^{-1}	0.75	2.17×10^{-1}	0.82
DOA rs86567	intron(A>C)	8.94×10^{-1}	0.96	2.42×10^{-1}	0.82	4.59×10^{-1}	0.86	8.30×10^{-2}	0.71	4.98×10^{-1}	0.85	1.28×10^{-1}	0.82
DOA rs2284191	intron(G>A)	4.10×10^{-2}	0.64	7.72×10^{-1}	0.85	5.00×10^{-2}	0.68	1.40×10^{-2}	0.24	6.83×10^{-1}	0.88	2.40×10^{-2}	0.41
DOA rs6913008	intron(C>T)	1.21×10^{-1}	0.64	2.28×10^{-1}	0.82	8.7×10^{-1}	0.90	3.99×10^{-1}	0.79	4.36×10^{-1}	0.78	6.43×10^{-1}	0.87
DOA rs2582	3'UTR(C>A)	7.87×10^{-1}	0.89	9.33×10^{-1}	0.96	8.38×10^{-1}	0.90	5.26×10^{-1}	0.81	7.76×10^{-1}	0.94	6.51×10^{-1}	0.87
DOA rs416622	3'UTR(G>A)	9.49×10^{-1}	0.96	3.75×10^{-1}	0.82	6.94×10^{-1}	0.88	3.00×10^{-3}	0.10	3.00×10^{-1}	0.75	1.02×10^{-1}	0.82
DOA rs453779	intron(C>T)	6.39×10^{-1}	0.86	7.03×10^{-1}	0.85	8.57×10^{-1}	0.90	4.32×10^{-1}	0.79	9.00×10^{-1}	0.99	5.19×10^{-1}	0.87
DOA rs1044429	3'UTR(G>A)	7.45×10^{-1}	0.89	6.73×10^{-1}	0.85	6.64×10^{-1}	0.88	9.18×10^{-1}	0.96	8.12×10^{-1}	0.94	8.57×10^{-1}	0.92
DOB rs2857111	intron(A>G)	6.54×10^{-1}	0.86	4.69×10^{-1}	0.82	8.96×10^{-1}	0.90	2.73×10^{-1}	0.72	3.45×10^{-1}	0.75	2.18×10^{-1}	0.82
DOB rs11244	3'UTR(C>T)	4.26×10^{-1}	0.80	7.30×10^{-1}	0.85	5.30×10^{-1}	0.86	5.19×10^{-1}	0.81	8.26×10^{-1}	0.94	5.43×10^{-1}	0.87
DOB rs1383258	intron(G>A)	1.36×10^{-1}	0.64	4.36×10^{-1}	0.82	2.63×10^{-1}	0.72	4.52×10^{-1}	0.79	6.30×10^{-2}	0.43	8.24×10^{-1}	0.92
DOB rs2071469	5'UTR(A>G)	1.92×10^{-1}	0.64	1.66×10^{-1}	0.82	1.21×10^{-1}	0.68	2.73×10^{-1}	0.72	3.75×10^{-1}	0.75	2.45×10^{-1}	0.83
DOB rs2071472	intron(G>A)	5.31×10^{-1}	0.83	4.81×10^{-1}	0.82	4.21×10^{-1}	0.86	8.61×10^{-1}	0.96	4.38×10^{-1}	0.78	5.98×10^{-1}	0.87
DOB rs7383287	synonymous(A>G)	9.61×10^{-1}	0.96	5.58×10^{-1}	0.82	8.00×10^{-1}	0.90	2.50×10^{-2}	0.28	1.00×10^{-2}	0.15	9.00×10^{-3}	0.31
DOB rs2856997	intron(T>G)	7.21×10^{-1}	0.89	4.42×10^{-1}	0.82	8.22×10^{-1}	0.90	2.44×10^{-1}	0.72	2.43×10^{-1}	0.75	1.50×10^{-1}	0.82
DOB rs2071471	synonymous(C>T)	7.87×10^{-1}	0.89	5.36×10^{-1}	0.82	6.96×10^{-1}	0.88	7.10×10^{-1}	0.91	6.62×10^{-1}	0.88	8.00×10^{-1}	0.92
DOB rs2071475	intron(C>T)	3.70×10^{-1}	0.74	8.50×10^{-1}	0.90	5.26×10^{-1}	0.86	2.16×10^{-1}	0.72	6.27×10^{-1}	0.88	4.45×10^{-1}	0.87
DOB rs2856995	intron(T>A)	3.11×10^{-1}	0.74	5.05×10^{-1}	0.82	2.98×10^{-1}	0.72	2.64×10^{-1}	0.72	9.81×10^{-1}	0.99	4.30×10^{-1}	0.87
TAP1 rs1135216	Missense(A>G)	3.53×10^{-1}	0.74	7.07×10^{-1}	0.85	5.00×10^{-1}	0.86	6.73×10^{-1}	0.91	3.18×10^{-1}	0.75	4.78×10^{-1}	0.87

SNPs	Location	Dominant		Recessive		Additive		Dominant		Recessive		Additive	
		P* Value	**FDR***	***P* Value***	**FDR***	***P* Value***	**FDR***	***P* Value†**	**FDR†**	***P* Value†**	**FDR†**	***P* Value†**	**FDR†**
TAP2 rs1800454	Missense(G>A)	5.52×10^{-1}	0.83	3.18×10^{-1}	0.82	8.30×10^{-1}	0.90	1.58×10^{-1}	0.72	5.59×10^{-1}	0.88	3.05×10^{-1}	0.84
LMP2 rs17587	Missense(G>A)	1.70×10^{-1}	0.64	2.55×10^{-1}	0.82	1.32×10^{-1}	0.68	3.03×10^{-1}	0.72	9.00×10^{-3}	0.15	7.70×10^{-2}	0.82
LMP7 rs2071543	Missense(C>A)	6.02×10^{-1}	0.85	4.75×10^{-1}	0.82	4.89×10^{-1}	0.86	7.49×10^{-1}	0.91	4.90×10^{-2}	0.42	3.18×10^{-1}	0.84
tapasin rs9277972	intron(A>T)	1.29×10^{-1}	0.64	7.48×10^{-1}	0.85	1.55×10^{-1}	0.68	9.79×10^{-1}	0.98	2.35×10^{-1}	0.75	6.97×10^{-1}	0.88
tapasin rs1059288	5'near gene(T>C)	2.07×10^{-1}	0.64	1.00×10^{-1}	0.82	8.2×10^{-2}	0.68	9.29×10^{-1}	0.96	6.84×10^{-1}	0.88	8.77×10^{-1}	0.92
tapasin rs2282851	intron(C>T)	9.03×10^{-1}	0.96	3.13×10^{-1}	0.82	6.10×10^{-1}	0.88	9.02×10^{-1}	0.96	9.51×10^{-1}	0.99	9.01×10^{-1}	0.92

Logistic regression analyses adjusted for age, gender, experience of blood/plasma donation, and numbers of blood/plasma donation.

* HCV infected cases (including both resolvers and chronic cases) versus uninfected subjects.

† HCV chronic cases versus HCV resolvers.

Supplemental Table 3. Conditional analyses of selected SNPs in additive genetic model

a) SNPs associated with anti-HCV status

SNPs	OR (95%CI)[a]	OR(95%CI)[b]	OR (95%CI)[c]
rs1063478	**0.77(0.62,0.97)** [d]	0.79(0.64,0.98)	0.78(0.63-0.98)
rs2284191	1.42(1.02,1.97)	**1.45(1.03,2.03)** [d]	1.41(1.00-1.97)
rs11244	1.23(0.97,1.56)	1.27(1.01,1.62)	**1.24(0.98-1.57)** [d]

b) SNPs associated with HCV chronicity

SNPs	OR (95%CI)[e]	OR(95%CI)[f]	OR (95%CI)[g]	OR (95%CI)[h]	OR (95%CI)[i]	OR (95%CI)[j]
rs17587	**0.71(0.52,0.97)**[k]	0.72(0.53,0.99)	0.77(0.57,1.04)	0.75(0.55,1.01)	0.77(0.57,1.05)	0.78(0.58,1.06)
rs2071543	0.77(0.58,1.04)	**0.80(0.59,1.08)** [k]	0.84(0.63,1.12)	0.86(0.64,1.15)	0.83(0.62,1.11)	0.83(0.62,1.10)
rs2284191	0.65(0.45,0.95)	0.66(0.45,0.96)	**0.71(0.46,1.10)** [k]	0.66(0.45,0.97)	0.65(0.45,0.95)	0.70(0.46,1.07)
rs7383287	0.72(0.58,0.92)	0.74(0.59,0.92)	0.73(0.59,0.92)	**0.74(0.59,0.92)** [k]	0.73(0.58,0.91)	0.74(0.59,0.92)
rs376892	0.97(0.73,1.30)	0.96(0.72,1.29)	0.98(0.73,1.32)	0.96(0.72,1.29)	**1.08(0.74,1.57)**[k]	1.17(0.82,1.67)
rs416622	0.83(0.65,1.06)	0.81(0.64,1.04)	0.90(0.69,1.18)	0.83(0.65,1.07)	0.76(0.56,1.02)	**0.89(0.63,1.27)** [k]

Logistic regression analyses adjusted for age, gender, experience of blood/plasma donation, and numbers of blood/plasma donation.
[a] Logistic regression analyses conditioned on rs1063478
[b] Logistic regression analyses conditioned on rs2284191
[c] Logistic regression analyses conditioned on rs11244
[d] Logistic regression analyses conditioned on the other two polymorphisms
[e] Logistic regression analyses conditioned on rs17587
[f] Logistic regression analyses conditioned on rs2071543
[g] Logistic regression analyses conditioned on rs2284191
[h] Logistic regression analyses conditioned on rs7383287
[i] Logistic regression analyses conditioned on rs376892
[j] Logistic regression analyses conditioned on rs416622
[k] Logistic regression analyses conditioned on the other five polymorphisms

Supplemental Table 4. Stratified analyses of combined favorable alleles and HCV infection outcomes

a) SNPs associated with anti-HCV status (rs1063478-T and rs2284191-G, 0-2 vs.3-4)

Variables	Uninfected	Infected	OR (95%CI)	*P*
Age				0.123
<60	152/142	250/159	0.62 (0.43-0.88)	
≥60	112/76	189/127	0.84 (0.53-1.34)	
Gender				0.023
Male	77/57	99/60	0.75 (0.41-1.38)	
Female	187/161	340/226	0.68 (0.49-0.94)	
Blood donation				0.971
Never	33/24	50/29	0.55 (0.24-1.28)	
Ever	231/194	389/257	0.69 (0.51-0.94)	
Numbers of blood donation				0.691
<30	137/113	234/159	0.79 (0.54-1.15)	
≥30	127/105	205/127	0.61 (0.40-0.93)	
Plasma donation				0.024
Never	80/64	18/25	1.14 (0.51-2.57)	
Ever	184/154	421/261	0.66 (0.49-0.90)	
Numbers of plasma donation				0.706
<50	199/164	251/156	0.67 (0.47-0.95)	
≥50	65/54	188/130	0.72 (0.45-1.16)	

b) SNPs associated with HCV chronicity (rs17587-A and rs7383287-G, 0 vs.1-4)

Variables	Resolver	Chronic	OR (95%CI)	*P*
Age				0.105
<60	38/73	154/144	0.49 (0.31-0.78)	
≥60	33/49	103/131	0.90 (0.53-1.51)	
Gender				0.923
Male	15/24	61/59	0.51 (0.23-1.13)	
Female	56/98	196/216	0.63 (0.43-0.93)	
Blood donation				0.332
Never	6/17	27/29	0.41 (0.13-1.36)	
Ever	65/105	230/246	0.67 (0.47-0.97)	
Numbers of blood donation				0.144
<30	33/78	127/155	0.50 (0.31-0.81)	
≥30	38/44	130/120	0.80 (0.48-1.33)	
Plasma donation				0.403

Variables	Resolver	Chronic	OR (95%CI)	***P***
Never	6/8	12/17	0.70 (0.09-5.35)	
Ever	65/114	245/258	0.61 (0.43-0.87)	
Numbers of plasma donation				0.167
<50	44/61	146/156	0.91 (0.57-1.46)	
≥50	27/6	111/119	0.46 (0.27-0.78)	

Logistic regression analyses adjusted for age, gender, experience of blood/plasma donation, and numbers of blood/plasma donation (excluded the stratified factor in each stratum).
[a] *P* value for the heterogeneity test.

Supplemental Table 5. Linkage disequilibrium (LD) information in uninfected subjects

a) LD of rs1063478, rs2284191, and rs11244

	rs1063478	rs2284191	rs11244
rs1063478	--	0.10 [a]	0.21[a]
rs2284191	0 [b]	--	0.04 [a]
rs11244	0.01 [b]	0 [b]	--

b) LD of rs17587, rs2071543, rs2284191, rs7383287, rs376892, and rs416622

	rs17587	rs2071543	rs2284191	rs7383287	rs376892	rs416622
rs17587	--	0.87 [a]	0.04 [a]	0.24 [a]	0.05 [a]	0.34 [a]
rs2071543	0.04 [b]	--	0.02 [a]	0.08 [a]	0.01 [a]	0.08 [a]
rs2284191	0 [b]	0 [b]	--	0.03 [a]	0.10 [a]	0.94 [a]
rs7383287	0 [b]	0.01 [b]	0 [b]	--	0.01 [a]	0.08 [a]
rs376892	0 [b]	0 [b]	0 [b]	0 [b]	--	0.82 [a]
rs416622	0 [b]	0 [b]	0.19 [b]	0 [b]	0.34 [b]	--

[a] D’; [b] R
